# Cell Invasion in the Spheroid Sprouting Assay: A Spatial Organisation Analysis Adaptable to Cell Behaviour

**DOI:** 10.1371/journal.pone.0097019

**Published:** 2014-05-07

**Authors:** Silvia Blacher, Charlotte Erpicum, Bénédicte Lenoir, Jenny Paupert, Gustavo Moraes, Sandra Ormenese, Eric Bullinger, Agnès Noel

**Affiliations:** 1 Laboratory of tumor and developmental biology, GIGA-Cancer, University of Liège, Liège, Belgium; 2 Laboratory of cardiovascular research, CRP santé, Luxembourg, Luxembourg; 3 GIGA-Imaging and Flow Cytometry Platform, University of Liege, Liege, Belgium; 4 GIGA Systems Biology and Chemical Biology, University of Liege, Liege, Belgium; Medical University Innsbruck, Austria

## Abstract

The endothelial cell spheroid assay provides a suitable *in vitro* model to study (lymph) angiogenesis and test pro- and anti-(lymph) angiogenic factors or drugs. Usually, the extent of cell invasion, observed through optical microscopy, is measured. The present study proposes the spatial distribution of migrated cells as a new descriptor of the (lymph) angiogenic response. The utility of this novel method rests with its capacity to locally characterise spheroid structure, allowing not only the investigation of single and collective cell invasion but also the evolution of the spheroid core itself. Moreover, the proposed method can be applied to 2D-projected spheroid images obtained by optical microscopy, as well as to 3D images acquired by confocal microscopy. To validate the proposed methodology, endothelial cell invasion was evaluated under different experimental conditions. The results were compared with widely used global parameters. The comparison shows that our method prevents local spheroid modifications from being overlooked and leading to the possible misinterpretation of results.

## Introduction

Angiogenesis and lymphangiogenesis refer to the formation of new blood and lymphatic vessels, respectively. They are associated with various pathological conditions such as cancer, metastatic dissemination, psoriasis, graft rejection and ocular disorders, among others [1]–[5]. These biological processes are characterised by a complex cascade of events, during which quiescent endothelial cells (ECs) become activated to degrade their surrounding extracellular matrix, directionally migrate towards the (lymph) angiogenic stimulus, proliferate and organise into new three-dimensional (3D) capillary networks [6]. Migrating blood and lymphatic ECs (BECs and LECs, respectively) are confronted *in vivo* by the basement membrane or interstitial matrix, which act as physical barriers against moving cells [3], [7], [8]. Consequently, different *in vitro* models have been developed to challenge ECs to 3D-reconstituted matrices of type I collagen, matrigel or fibrin [2], [3], [9]–[11].

Among classical angiogenesis models, the spheroid sprouting assay consists of the self-aggregation of ECs embedded in a 3D matrix leading to EC sprouting and invasion into the surrounding matrix. This latter situation perfectly reproduces the formation of capillaries from pre-existing vessels. This 3D-gel-embedded EC spheroid model has gained broad acceptance due to its numerous advantages. Indeed, it i) provides a better mimic of the *in vivo* environment than classical 2D-cultures, ii) is rapid and easy to use, iii) takes into account different cell properties involved in angiogenesis (e.g., cell proliferation, migration, invasion, survival), and iv) lacks inflammatory complications and thereby facilitates the investigation of cellular and molecular mechanisms underlying angiogenesis. In addition, defined experimental conditions can easily be achieved to facilitate screens for pro- or anti-angiogenic agents and to evaluate the impact of biochemical and/or physical barriers on cell invasion [10], [12]–[14].

When we conducted experiments aimed at challenging this assay, we observed that cell motion can give rise to different organisations of not only the migrating cells but also the spheroid bulk itself, depending on the experimental conditions. Indeed, several different cell behaviours are seen: (i) cells can move as groups of cells (collective invasion) or as single cells (individual invasion); (ii) cells can remain connected to or detach from the spheroid core; and (iii) in the spheroid itself, the extent of cell aggregation can vary (spheroid retraction or expansion). To date, no method has been available to quantitatively analyse the different cell behaviours that drive EC sprouting and morphogenesis.

Measurements of EC migration assay images are usually performed using manual methods, which leads to the global characterisation of structures without regard for the specific features of the spheroid and the migrating ECs. Currently, most researchers either determine the cumulative length of outgrowing capillaries using an ocular grid [13], [15], [16] or count isolated cells [17]. Semi-automatic and automatic methods have also been developed to determine global descriptors such as the total area covered by cells, factor shape and the fragmentation degree of the spheroids, as well as the maximal distance of migration, the number of vessel and cumulative vessel length [18], [19]. Despite their undeniable utility, these global measurements are unable to detect precise modifications of cell behaviour and/or organisation. Notably, identical total spheroid areas or maximum migration distances could be obtained from ECs with different behaviours at the cellular level in terms of invasion, tube formation and branching.

In this work, the evaluation of the spatial EC density distribution is proposed for the quantitative, in-depth investigation of (lymph) angiogenesis in the spheroid assay. It is argued that this cell distribution determination enables the detection of modifications in the extent of cell aggregation in the spheroid core and underlines the different modes of cell invasion as a function of the experimental conditions. To highlight the potential appeal of this new descriptor, EC spheroids have been subjected to different collagen matrices in the presence or absence of inhibitors. Using these experiments, the proposed methodology, as well as classical methods used to characterise 2D-projected images of spheroids obtained from optical microscopy, were investigated. The 3D generalisation of the proposed methodology was then applied to 3D spheroid images obtained via confocal microscopy.

## Materials and Methods

### LEC Culture, Collagen Preparation and Spheroid Assay

Human telomerase-transfected dermal LECs (hTERT-HDLECs) [20] or human microvascular LECs (hMVEC-dly, Lonza, Invitrogen) were grown in EGM2-MV medium (Lonza, Invitrogen). The specific MMP (matrix metalloproteinase) 2 inhibitor and PMA (phorbol myristate acetate) were purchased from Calbiochem (Darmstadt, Germany) and Sigma (Saint-Louis, USA), respectively. The broad-spectrum MMP inhibitor RO-28-2653 was used as previously described [21].

For 3D cell cultures, two collagen preparations were used: (1) pepsin-extracted type I collagen (“pepsinized collagen”) (Rat tail collagen High Concentration, Type1, BD Biosciences, MA, USA), and (2) telopeptide-intact collagen (“native collagen”) extracted from rat tail tendons [7], [22]. The collagen preps were solubilised at the desired concentration in 0.1% glacial acetic acid. To generate multicellular spheroids, hTERT-HDLECs or hMVEC-dly cells were seeded in EBM-2 medium containing 0.24% high viscosity methyl cellulose (Sigma Aldrich, Saint Louis, MO) (2×10^3^ cells per well) [18]. After 24 h of culture, the spheroids were collected, mixed with collagen gels and immediately seeded on a thin layer of collagen gel as previously described [18]. After 24 h or 48 h of culture in EGM2-MV medium, cell invasion was visualised using a ZEISS Axiovert 25 microscope at 10x magnification. At least 10 spheroids were analysed per experimental group.

### Confocal Image Acquisition

For spheroid immunochemistry, cultures were grown in Vision Plate 4titude (Bioké, Leiden, The Netherlands). Whole spheroids were fixed in paraformaldehyde (1%). After washes and permeabilisation with Triton X-100 (0.1%), spheroids were immunolabelled with phalloidin coupled to Atto 550 (Sigma-Aldrich, Schnelldorf, Germany) (1/500). Nuclei were stained with Vectashield Dapi (Molecular Probe, Merelbeke, Belgium). The 3D spheroids were visualised under a Nikon A1R laser scanning confocal microscope (Nikon Instruments Inc, Japan). Images were obtained at 20x magnification (Plan Apo 20x ELWD, NA 0.8, WD 1000 µM) using two lasers (405 nm and 561 nm, in sequential mode) and the emission filters 450/25 and 595/25 for DAPI and DsRed, respectively. Nikon NIS Elements Advanced Research software (V4.0) was used during all image acquisition procedures. The whole Z dimension of each spheroid was scanned with an optical section thickness of 5,79 µm (1,2 airy units) and an interval between sections of 3 µm. Images of 1024×1024 pixels (12 bit) were obtained with an XY optical resolution of 0,24 µm. The 3D images from each assay were then built by stacking approx. N = 100 cross sections.

### Cell Invasion and Image Processing

Image characteristics depend strongly on experimental conditions (cell types, motion, collagen substrate, etc.) and on the image acquisition technique (type of microscope, as well as observation parameters such as contrast, illumination, magnification, etc.). In this section, we describe the image processing utilised in this work. Note that the different steps need to be carefully adapted for each application.

### 2D-projected Images of the Spheroid Assay

**Figure 1:**
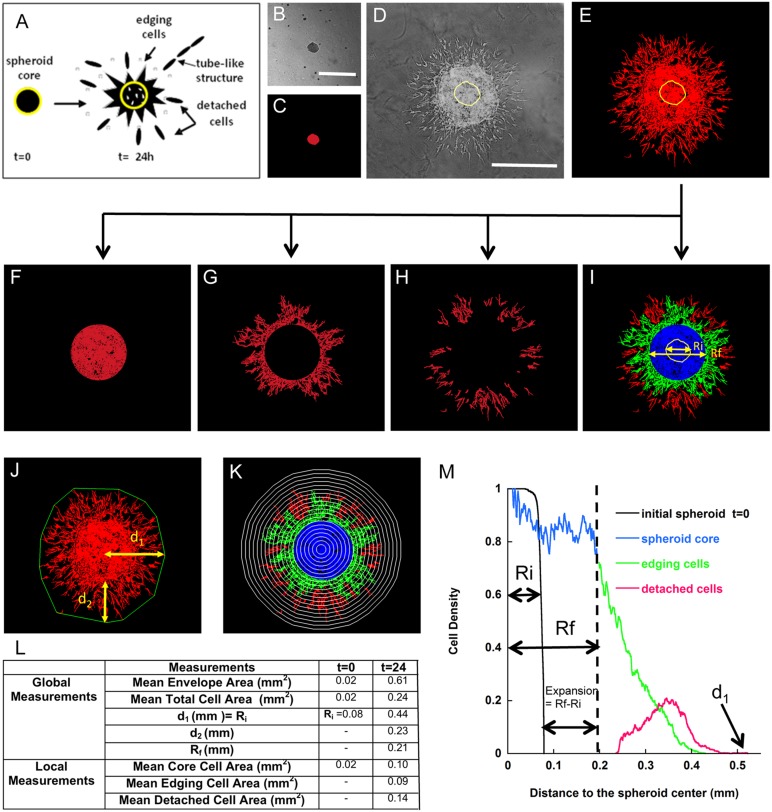
Description of the spheroid assay and the method of quantification. (a) Schematic representation of spheroid evolution during cell culture. The initial spheroid is shown on the left (yellow circle) and the different modes of cell sprouting and invasion are depicted on the right panel after 24 h of culture. (b–e) Representative pictures of spheroids after embedding into the collagen matrix (t = 0 h, b) and after 24 h of culture (t = 24 h, d), along with their corresponding binarised images (c, e). (f–h) Decomposition of the binarised image into three components: spheroid core (f), edging cells (g) and detached cells (h). (i) Representation of the whole spheroid and its components: the initial spheroid delineated by a yellow circle, the expanded spheroid core (blue), edging cells (green) and detached cells (red). (j) Illustration of the parameters used for global measurements: convex envelope (green) and total distance of cell invasion starting from the spheroid centre (d_1_) or border (d_2_). (k) Grid used for local measurements: a circular grid is superimposed on the coloured spheroid representation. (l) Comparison of global and local measurements at t = 0 and t = 24 h. (m) Graph representing the cell density distribution measured from the image. The colours of the curves correspond to the different spheroid components described in the other panels (a, i and k). Bars = 500 µm.

When embedded in a collagen gel, cells can adopt different modes of invasion as depicted in Figure 1 a. During invasion, cells can remain linked to the spheroid, leading to the generation of a rough spheroid, spread out from the spheroid or detach from it to migrate as single cells or aligned in tube-like structures. This cell invasion process can lead to empty regions in the core spheroid. Image processing prior to image measurement classically consists of filtering the images to eliminate noise and increase the contrast followed by image binarisation, in which objects of interest, in this case cells, are assigned a pixel value of 1, with the background assigned a value of 0. To achieve this, we proceeded in several steps (Figure 1). At t = 0, the spheroid image was usually very well contrasted (Figure 1 b), and the choice of an appropriate threshold for binarisation was straightforward (Figure 1 c). At later time points (t>0), the spheroid consisted of an expanded spheroid core and migrating cells (Figure 1 d). These features were discriminated using the Frangi multiscale filter [23] (Figure 1 e), which allows for the identification of a wide range of object sizes, elongations and orientations in images presenting non-homogeneous intensities. In some cases, morphological filters [24] were used to eliminate any remaining noise in the images.

Using morphological and logical operators [24], the resulting binary image (Figure 1 e) was decomposed into three components: (i) the spheroid core, composed of the cells forming the initial spheroid (Figure 1 f), (ii) the edging cells, herein referred to as migrating cells, still attached to the spheroid core (Figure 1 g), and (iii) the detached cells, which are shed from the spheroid and migrate as single cells or as tube-like structures (Figure 1 h). The smooth frontier between the core spheroid and the border cells was defined as the largest inscribed circle, including internal regions without cells, i.e., visualised as gaps not connected to the exterior (Figure 1 f). The so-defined frontier allowed the core spheroid to be distinguished from border cells (Figure 1 g).

### 3D Images of the Spheroid Assay

3D spheroid images obtained by confocal microscopy consisted of stacks of up to approximately 100 colour RGB (Red Green Blue colour space) images of 1024×1024 pixels, in which the red component corresponds to cells and the blue component to the nucleus. In this work, only the red component of each slice was considered. The processing of each 3D image as a single object is hampered by the requirement for substantial amounts of computer memory, its time consumption and the difficulty of ensuring the correctness of the final cell segmentation at each processing step. For this reason, image transformation and segmentation were performed sequentially on neighbouring slices. A median (3×3) low-pass filter [25] was first applied to the original slices to eliminate random impulse noise currents in the confocal images. The filtered images were then automatically binarised using the Otsu method [26]. Next, small spurious objects were eliminated, keeping only those appearing in at least three neighbouring slices, i.e., larger than a 5-µm cell size. Finally, the 1024×1024×N images were resized to accelerate the calculations and to facilitate 3D image visualisation. To this end, the maximum intensity in non-overlapping blocks of 2×2×2-sized voxels was calculated. Those values become a single pixel in a new 512×512× (N/2)-sized image.

2D and 3D image processing and measurements were implemented using the image analysis toolbox of Matlab7.9 (MathWorks) software.

## Results

### Analyses of Cell Invasion and Spheroid Core Modification in the 2D Projection of the Spheroid Assay

Angiogenesis was explored in LEC spheroids seeded in collagen gels and cultured for different times. LECs originating from the spheroids progressively invade the gel and migrate either individually as unicellular sprouts (single cells) or collectively as complex capillary-like structures (Figure 1 a). Three cellular components were distinguished in the spheroid assay as described in the Material and Methods: “the spheroid core”, “the edging cells” and “the detached cells” (Figure 1 a). In addition to cell invasion, morphological modifications in the spheroid core itself were detected. While the spheroids initially (t = 0, Figure 1 b) appeared as a compact structures, later (t>0) cell movement induced spheroid expansion and led to breaches in the spheroid core (Figure 1 d). To quantitatively analyse the changes in spheroid morphology and cell distribution, global and local measurements were performed.

To extract global parameters from the binary images, we first calculated the parameters most commonly presented in the literature (Figure 1 j): (i) the envelope area, defined as the area of the minimal convex polygon containing the whole spheroid; (ii) the envelope radius, “d_1_”; (iii) the distance, “d_2_”, between the spheroid border and the maximal point reached by the migrating cells; and (iv) the total area occupied by the spheroid and the migrating cells (total cell area). In addition, novel parameters were measured for the area covered by each of the aforementioned components of the spheroid: the spheroid core area (Figure 1 f), the edging cell area (Figure 1 g) and the detached cell area (Figure 1 h).

Because the spheroid model presents a radially symmetrical geometry, the simplest morphological characterisation to determine the spatial cell distribution is the evaluation of the spatial distribution of cells around the initial cell aggregate. Considering a set of circles (i) of growing radius (d_i_) and perimeter P_i_ centred at the centre of the original spheroid (t = 0), the local *cell density* is defined as the number of pixels belonging to cells that intersect the circle “i” (N_i_), normalised by the corresponding perimeter P_i_ (Figure 1 k). Then, the spatial cell density distribution is represented by a graph of the cell densities as a function of increasing radius (d_i_) (Figure 1 m). The table (Figure 1 l) determined from the image represented in Figures 1 i and j compares the distributions at t = 0 and t = 24 h. After 24 h of culture, cell invasion is described by two cell distribution curves. The first curve corresponds to the spheroid core and the attached migrating cells (the edging cells). The second bell-shaped curve reflects the distribution of the detached migrating cells. The superimposition of the edging and detached cell distribution curves highlights the spatial coexistence of the attached and detached cells. Notably, the previously described global parameters, i.e., the total area occupied by the spheroid or by each spheroid component, can be calculated from the density cell distribution curve by determining the area under the corresponding portion of the curve. In addition, the maximum value reached on the distance axis by the cell density distribution curve gives the maximum distance reached by the migrating cells (“d_1_”) (Figure 1 k). In the example shown, a comparison of the initial spheroid and the core spheroid at the end of the assay reveals a potential spheroid expansion, as illustrated in Figure 1 m, which results from collective outward cell motion. Indeed, similar results were generated in the presence of mitomycin C, excluding the impact of cell proliferation (data not shown). This spheroid expansion can be easily measured by subtracting the initial spheroid radius (r_i_) from the final spheroid radius (r_f_). This observation underscores the importance of acquiring an image of the initial spheroid embedded in the matrix prior to culturing.

It is worth to noting that the total area of the spheroid reached 0.22 mm^2^, whereas the envelope area was 0.59 mm^2^, which clearly shows that this latter parameter overestimates the effects of invasion. Moreover, in the present case, the increased spheroid area is caused not only by cell invasion but by the expansion of the spheroid core, a process completely overlooked by the envelope area parameter.

### 2D Quantitative Assessments of Cell Invasion and Spheroid Core Modifications under Various Experimental Conditions

**Figure 2:**
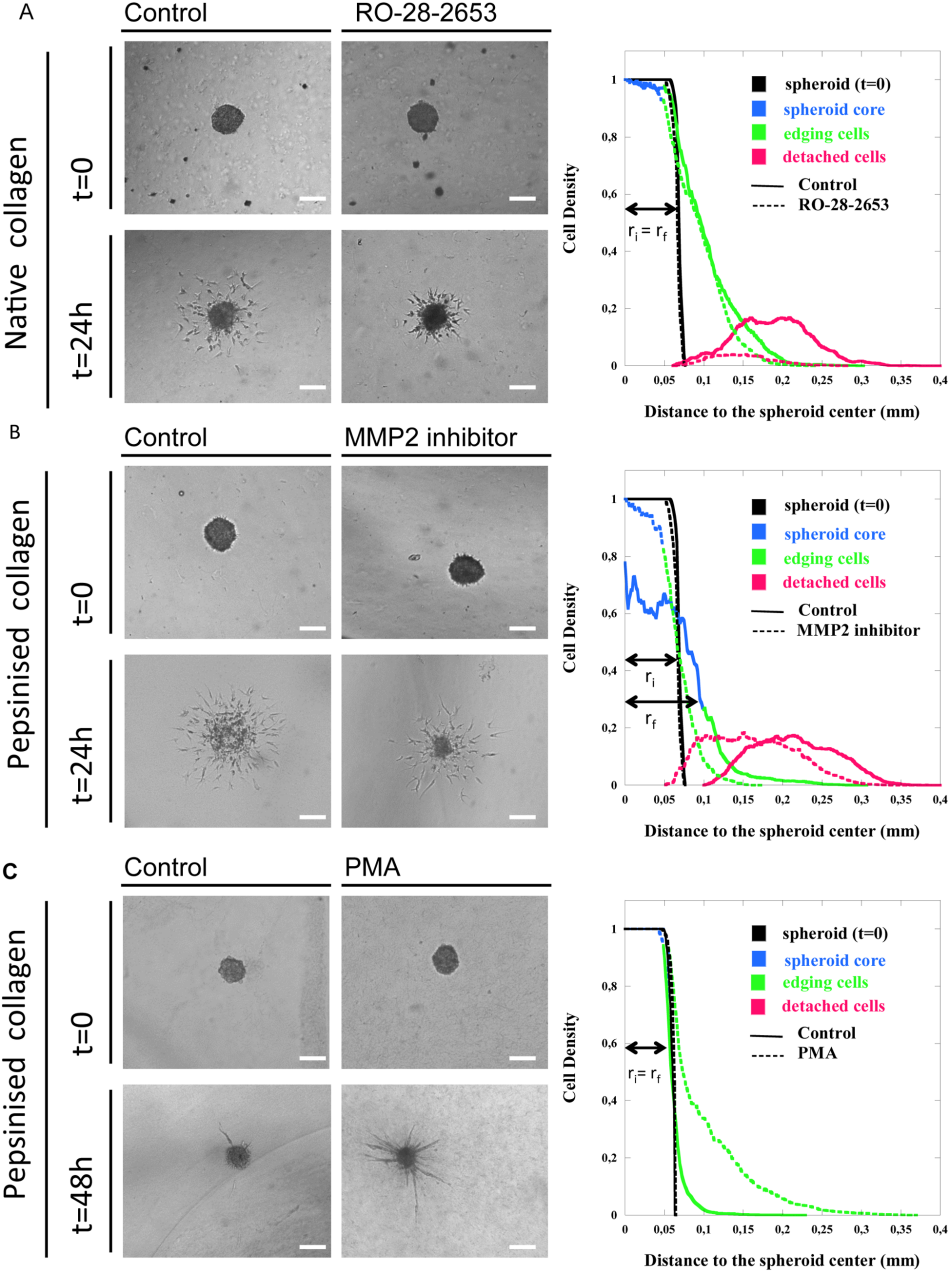
Spheroid assays in different collagen matrices. Two types of lymphatic endothelial cells, hTERT-HDLECs (a, b) and hMVEC-dly cells (c), were embedded in native collagen (2 mg/ml) (a) or in pepsinized collagen at a low (1,5 mg/ml) (b) or high concentration (2 mg/ml) (c). Cells were cultured in the absence (control) or presence of MMP-inhibitors (RO-28-2653 or MMP2 inhibitor) for 24 h (a, b) or a stimulator (PMA) for 48 h (c). For each assay, the initial spheroid (0 h) and the spheroid at the end of the assay (24 h or 48 h) are shown. Graphs on the right represent the density cell distributions measured around the spheroids. For clarity, the cell density distribution curves for each assay were rendered in three colours (blue, spheroid core; green, edging cells; red, detached cells) as in Figure 1. r_i_ and r_f_ correspond to the radius of the initial and final spheroid, respectively. Bars = 500 µm.

In order to validate the proposed methodology, three independent assays were conducted to explore the specific behaviours of immortalised (hTERT-HDLEC) and primary (hMVEC-dly) LECs. Due to the emerging importance of matrix stiffness and structure in pathological conditions, spheroids were embedded in different type I collagen matrices (pepsinized or native collagen). Inhibitors of MMPs, which are recognised as key modulators of cell invasion, were also used. Individual cell sprouting and invasion were first explored in spheroids embedded in a dense native collagen matrix in the presence or absence of a broad-spectrum MMP inhibitor (R0-28-2653) (Figure 2 a). Spheroid expansion concomitant to cell invasion was examined in a pepsinized collagen matrix following specific inhibition of MMP2 (Figure 2 b). Finally, the formation of capillary-like structures attached to the spheroid core was studied by embedding spheroids of poorly migrating hMVEC-dly cells in a pepsinized collagen matrix under phorbol ester (PMA) stimulation (Figure 2 c). For each assay, we compared classically used global parameters (envelope area, total cell area, d_1_ and d_2_; referred as global measurements in Tables 1 to 3) to spheroid component parameters (core spheroid area, edging cell area, detached cell area). In addition, the cell density distribution was also determined for each assay.

**Table 1:** Global and local measurements of spheroid components upon treatment with a broad-spectrum MMP inhibitor.

Measurements		t = 0	Control	RO-28-2653	Inhibition (%)
			(t = 24 h)	(t = 24 h)	
**Global**	**Mean Envelope Area (mm^2^)**	0.0182±0.0048	0.2223±0.0161	0.0914±0.00098****	59
**Measurements**	**Mean Total Cell Area (mm^2^)**	0.0182±0.0048	0.0643±0.0052	0.0360±0.0020****	44
	**d_1_ (mm)**	0.0760±0.0010	0.3332±0.0186	0.2499±0.0505**	25
	**d_2_ (mm)**	−	0.2673±0.0181	0.1906±0.0500**	29
**Local**	**Mean Core Cell Area (mm^2^)**	0.0182±0.0048	0.0144±0.0011	0.0102±0.0005**	30
**Measurements**	**Mean Edging Cell Area (mm^2^)**	−	0.0249±0.0026	0.0222±0.0012	ns
	**Mean Detached Cell Area (mm^2^)**	−	0.0250±0.0020	0.0036±0.0009*****	86

Lymphatic hTERT-HDLEC spheroids were embedded in a native collagen matrix with or without (control) an MMP inhibitor (RO-28-2653) for 24 h. The parameters measured are those determined through the assay illustrated in Figure 2a.

**P<0,01;

****P<0,0001;

*****P<0,00001 (inhibitor versus control).

**Table 2:** Global and local measurements of spheroid components upon treatment with an MMP2 inhibitor.

Measurements		t = 0	Control	MMP2 inhibitor	Inhibition (%)
			(t = 24 h)	(t = 24 h)	
**Global**	**Mean Envelope Area (mm^2^)**	0.0149±0.0005	0.267±0.008	0.235±0.01*	12
**Measurements**	**Mean Total Cell Area (mm^2^)**	0.0149±0.0005	0.059±0.004	0.038±0.002****	36
	**d_1_ (mm)**	0.070±0.001	0.342±0.007	0.324±0.009	ns
	**d_2_ (mm)**	−	0.241±0.009	0.276±0.01*	27
**Local**	**Mean Core Cell Area (mm^2^)**	0.0149±0.0005	0.018±0.001	0.007±0.0004****	53
**Measurements**	**Mean Edging Cell Area (mm^2^)**	−	0.010±0.001	0.011±0.001	ns
	**Mean Detached Cell Area (mm^2^)**	−	0.031±0.002	0.027±0.001	ns

Lymphatic hTERT-HDLEC spheroids were embedded in a pepsinized collagen gel with or without (control) an MMP inhibitor (MMP2 inhibitor) for 24 h. The parameters measured are those determined through the assay illustrated in Figure 2b.

*P<0,05;

****P<0,0001 (inhibitor versus control).

**Table 3:** Global and local measurements of spheroid components upon treatment with PMA.

Measurements		t = 0	Control	PMA	Stimulation (%)
			(t = 48 h)	(t = 48 h)	
**Global**	**Mean Envelope Area (mm^2^)**	0.0126±0.0004	0.0184±0.0011	0.1459±0.0138****	87
**Measurements**	**Mean Total Cells Area (mm^2^)**	0.0126±0.0004	0.0127±0.0006	0.0386±0.0035****	67
	**Mean Total Cells Volume (mm^3^)**	0.0019±0.0001	0.0022±0.0002	0.0036±0.0003**	39
	**d_1_ (mm)**	0.0631±0.0011	0.1293±0.0123	0.3311±0.0113****	61
	**d_2_ (mm)**	−	0.0811±0.0011	0.2760±0.0012***	71
**Local**	**Mean Core Cell Area (mm^2^)**	0.0126±0.0004	0.0074±0.0004	0.0096±0.0004	ns
**Measurements**	**Mean Core Cells Volume (mm^3^)**	0.0019±0.0001	0.0022±0.0002	0.0021±0.0001	ns
	**Mean Edging Cells Area (mm^2^)**	−	0.0053±0.0004	0.0291±0.0038***	82
	**Mean Edging Cells Area (mm^3^)**	−	−	0.0014±0.0001	−

Lymphatic hMVEC-dly spheroids were embedded in a pepsinized collagen gel with or without (control) PMA for 48 h. The parameters measured are those determined through the assay illustrated in Figure 2c.

**P<0,01;

***P<0,001;

****P<0,0001 (inhibitor versus control).

In one assay (Figure 2 a), hTERT-HDLECs were embedded in a dense collagen matrix composed of native collagen (2 mg/ml) (t = 0) with or without RO-28-2653 treatment. The increase in the global parameters observed after 24 h of culture (t = 24 h) (Table 1) indicates that the cells spread out in both experimental conditions. However, upon RO-28-2653 treatment, the envelope area was 59% smaller than in the control conditions. This parameter overestimates the inhibitory effect compared to the measurement of the total area occupied by the cells, which reveals a 44% inhibition of cell invasion (Table 1). The distances of cell invasion, measured either from the spheroid centre (d_1_) or from the spheroid border (d_2_), were 25% and 29% smaller upon MMP inhibition, respectively. The spatial cell density distributions highlighted specific features of each spheroid component (Figure 2 a). For both control and treated spheroids, the mean core cell area measured after 24 h of culture was lower than the initial value (t = 0). This reduction in spheroid size was higher upon inhibitor treatment (Table 1). As the spheroid core remained almost compact, this suggests that cell invasion occurred from the surface of the spheroid such that its size decreased as invasion progressed.

Equivalent amount of edging cells remained attached and close to the spheroid in both experimental conditions. Remarkably, the detached cell area was reduced by 86% upon inhibitor treatment (Table 1), indicating that only a few isolated cells succeeded in detaching from the cell aggregate (Figure 2 a). These observations demonstrate that the inhibitor prevented cell detachment from the spheroid and almost preserved the initial spheroid structure.

In a second assay (Figure 2 b), hTERT-HDLECs were embedded in a matrix composed of pepsinized collagen (1,5 mg/ml). Due to the reduced physical constraints compared with the native collagen used above, after 24 h of culture the spheroid adopted an unpacked structure clearly reflecting the expansion of the initial spheroid (Figure 2 b). In sharp contrast, the core spheroid kept its original shape and even decreased in size upon MMP2 inhibitor treatment (Figure 2 b). Global measurements revealed a slight reduction in the envelope area (12%) and a greater reduction in the total cell area (36%). This again supports the strength of the total cell area measurement compared to the envelop determination. Surprisingly, the distance d_1_ was comparable between the two conditions, whereas the distance d_2_ was significantly larger (27%) upon treatment with the inhibitor (Table 2). Quantification of the spheroid components shed light on the invasion process and provided an explanation for the seemingly conflicting data (Table 2). After 24 h of culture, the mean core area was larger in the controls (0.018±0.001 mm^2^) and was almost halved upon inhibitor treatment (0.007±0.0004 mm^2^) compared to the initial spheroid (0.0149±0.0005 mm^2^) (Table 2).

The spatial cell distribution (Figure 2 b) revealed that the mean core density was reduced by 50% in the control condition and by only 5% upon inhibitor treatment. These results reflect the expansion of the control core spheroids during the 24 h of culture, whereas a reduction in core spheroid size was detected upon MMP inhibition. These data also explain why the distances d_1_ and d_2_, which disregard core reduction or expansion, failed to detect differences in cell invasion. Therefore, global parameters such as the distance of invasion d_1_, d_2_ or the envelope area fail to detect these local behaviours and could therefore lead to the misinterpretation of the results.

In a third assay (Figure 2 c), hMVEC-dly-cell spheroids embedded in pepsinized collagen (2 mg/ml) were cultured for 48 h in the presence of PMA to stimulate cell invasion. Under these conditions, cells sprouted outwards without detaching from the spheroid core and organised into capillary-like structures radiating around the spheroid core. The mean envelope area was 87% larger in the PMA-treated samples than in the control conditions (Table 3), whereas the total area occupied by cells and the “d_1_” distance increased by 61 and 67%, respectively (Table 3). The spatial cell density distributions (Figure 2 c) revealed that the spheroid cores tended to decrease in size (Table 3). This suggests that cell invasion began through the movement of cells from the spheroid surface. In stimulated samples, edging cells migrated collectively, pushing each other and organising as aligned cells. In sharp contrast, cells remained crowded near the spheroid core in control cultures. The maximum distance reached by sprouting cells in the capillary like structures (d_2_) was 71% higher in stimulated samples than in controls (Figure 2 c and Table 3). Importantly, no cells were able to detach from the spheroid and migrate individually, revealing the morphogenic impact of the treatment on ECs. It is worth noting again that the mean envelope area overestimates the differences observed between the experimental conditions because the presence of a very few long capillaries potentially introduces a bias in the envelop design and the measurement of its area.

### Cell Invasion and Spheroid Core Modification in 3D Images of the Spheroid Assay

**Figure 3:**
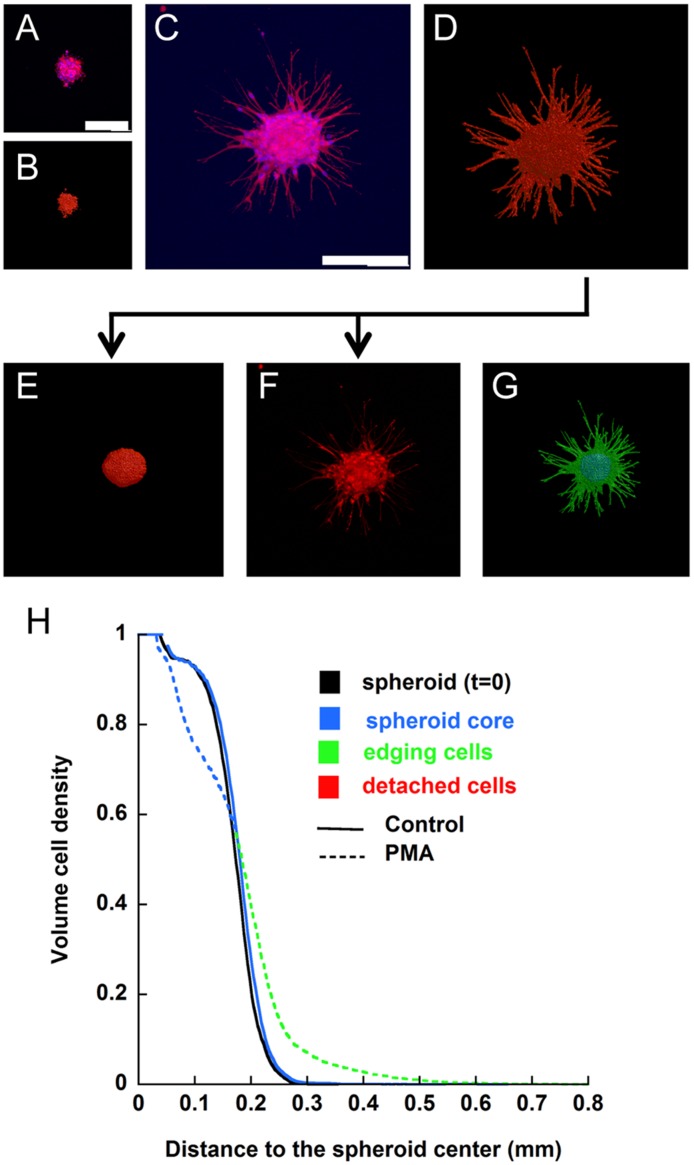
Description of 3D spheroid quantification. (a–d) Representative pictures of immunolabelled spheroids at t = 0 (a) or after 48 h of culture (c) and their corresponding binarised images (b, d). (e–g) Decomposition of the binarised image into the spheroid core (e) and edging cells (f). (g) Representation of the whole spheroid and its components, the spheroid core (blue) and the edging cells (green). (h) Graph representing the cell density distribution measured from the image. Bars = 500 µm.

The 2D quantification method was then generalised to enable its application to 3D images of the spheroids (Figure 3). To this end, we used PMA-treated spheroids. The 3D spheroid pictures (Figure 3 a and c) and their corresponding binarised images (Figure 3 b and d) show a complex organisation in which the thinnest vessel-like structures were not detected in the 2D projected images (Figures 2 c). It is worth noting that the entire spheroid is required for an accurate quantification of cell invasion from the centre and/or the border of the core. Interestingly, confocal microscopy enables the observation of the whole spheroid at 20x magnification, whereas 10x was the maximum magnification capable of capturing the entire object.

The spheroid core and the edging cells were identified using the same criteria as above (Figures 3 e–g). Notably, the spheroid core corresponded to an ellipsoid instead of a circle, and hence, the 3D spatial cell distribution was determined from a set of ellipsoids centred on the spheroid. Global and local measurements performed on the 3D images followed the same trend as those performed on the 2D projected images. However, the effects of PMA appeared more pronounced in the 3D analysis (Figures 2 c and 3 h).

The 3D distribution curves decreased by approximately d = 0.04 mm for both the initial (t = 0) and control spheroids (Figure 3 h). This decrease underlined the surface roughness of the spheroids, whose appearance was smooth in the 2D-projected images. On the other hand, the cell density volume decreased abruptly in the PMA-stimulated spheroids, indicating that cell invasion led to internal gaps without any contraction of the spheroid, thereby conserving its initial volume (Table 3). This effect was not detected in the 2D-projected images, which flatten the 3D image and hide the internal structure of the spheroid, making it appear as a compact object.

Finally, a large distance of cell invasion was measured in 3D space, suggesting that the thinnest and most distant tube-like structures, as well as adjacent vessels that appeared as joined structures, were not detected by standard optical microscopy.

## Discussion

The (lymph) angiogenic process is now widely viewed as a complex morphogenetic event that includes defined steps of cell specification, invasion, capillary organisation, tubular branching, network formation and maturation [6]. Among a number of 2D and 3D assays that have been developed, the EC spheroid-based in vitro (lymph) angiogenesis assay has become a widely used system to study (lymph) angiogenesis and its mechanisms [7], [10], [12]–[14], [18], [27], [28]. By utilising defined numbers of ECs and standardised experimental conditions, this method has proved to be both simple and highly reproducible. An important advantage of these cell aggregate-based angiogenesis assays is the possibility to confront spheroids with different 3D-matrices to better mimic angiogenic invasion as it occurs during angiogenesis in vivo. We proposed a new method for computerised image analysis to determine the extent of endothelial cell invasion, taking into account the particular structure of the 3D-matrix.

This proposed method was first validated on 2D-projected images obtained via optical microscopy. We have applied the EC spheroid assay to the analysis of the lymphangiogenic capacity of hMVEC-dly cells and hTERT-HDLECs, two lymphatic cell populations with distinct features. Thanks to their spontaneous ability to sprout from spheroids in complete culture medium, hTERT-HDLECs are highly suitable for evaluating the efficacy of inhibitors. In contrast, hMVEC-dly cells have a low migrative potential and require stimulation in order for lymphangiogenesis to occur. This makes them suitable target cells for the analysis of lymphangiogenesis-promoting substances.

Measuring the spatial cell density distribution quantifies cell density at all distances from the centre of the spheroid. The originality of this measurement relies on separating cells into different spheroid components, i.e., the core spheroid, the edging cells and the detached cells. This provides an intuitive insight into the spatial organisation of the cells, as well as into the often-overlooked spheroid transformation. First, we provide evidence that this method is powerful enough to quantify the invasion of ECs as single cells, as irradiating tube-like structures or as a complex anastomosed network attached to the initial spheroid. Second, a pharmacological approach illustrates the suitability of this method to evaluate the efficacy of inhibitors or stimulators of (lymph) angiogenesis. Third, our study highlights the fact that cell outgrowth from the spheroid is accompanied by modifications to the spheroid itself that are typically overlooked and might affect the quantification of invasion. The determination of the EC density as a function of the distance to the spheroid allows more information to be obtained regarding spheroid behaviour in different matrices. After the detection of the three regions, distribution measurements allow the accurate determination of the spheroid radius and the distance between the spheroid border or centre and the EC front. Our study underscores the importance of capturing an image of the initial spheroid in order to check the putative expansion of the spheroid that can occur in 3D-matrices that impose minimal physical constrains on the cells. We found that misleading results can be generated by determining the distance, d_2_, separating the migrating cell front and the spheroid border. Indeed, this parameter does not take into account putative spheroid expansion. Inversely, the distance d_1_ measured from the spheroid centre might also overestimate cell invasion depending on cell aggregate behaviour. Our data also demonstrate that the envelope covering the area of cell invasion is a parameter to be used cautiously because the presence of a very few highly migrative cells and/or long capillaries might bias its determination. The limitations of these widely used parameters necessitate caution when interpreting findings. Since no single parameter reflects all features of cell invasion, it is advisable to consider several parameters to get a better picture of the process. The spatial cell density distribution appears to be more appropriate for taking into account all of the cellular components that can be detected.

The proposed method of 2D image quantification was successfully generalised to 3D images. To the best of our knowledge, this is the first visualisation and computerised quantification of 3D spheroids. The results obtained by measuring the effects of PMA on spheroids indicated that the 2D and 3D measurements followed the same trends. However, the 3D measurements quantify core modifications in relation to the initial spheroid (t = 0) and reveal a larger extent of invasion, reflecting the detection of the thinnest tube-like structures.

Analyses of 2D and 3D images have their own advantages and drawbacks. 2D optical images of the whole spheroid are more easily acquired and allow the study of how cells migrate (individually or collectively), as well as the extent of cell invasion at the chosen magnification. However, these optical images are a 2D projection of a 3D object. This leads to two major limitations: the projection hides the true cell organisation, and the use of a high magnification introduces focusing artefacts (blurring) into the image, preventing the detection of the thinnest elongated cells and/or tube-like structures.

3D visualisation using confocal microscopy provides a more realistic picture of spheroid organisation since it is possible to work at higher magnifications without affecting image quality. It also enables the visualisation of cell invasion at different angles and can reveal the inner structure of the spheroid core. However, confocal image acquisition is not straightforward and requires sample staining and fixation, which is incompatible with time-lapse observations. Furthermore, 3D image quantification is both computationally demanding and very time consuming. Taking into account these issues and knowing that the analyses require almost 10 images per condition, we advise the use of this 3D technique in only those situations that require more precise quantification to provide deeper insights into the studied phenomena.

The idea that the local microenvironment, including the extracellular matrix, plays an important role in regulating cell behaviour has become increasingly accepted in cancer biology [29]. The attention of researchers has recently been focused on type I collagen as the main constituent of the interstitial matrix surrounding cancer cells [30] and migrating ECs [3], [31]. The precise architecture of the collagen gels used for in vitro assays is now recognised as an influential parameter of cell invasion [32]. When reconstituted from pepsin-extracted type I collagen, the absence of non-helical telopeptides situated at the N- and C-terminal ends of collagen molecules affects fibrillogenesis and the formation of collagen cross-links. The stiffness of matrices prepared from pepsinized collagen differs from that of gels formed from native collagen. In line with these considerations, cell detachment from the spheroid was detected in pepsinized collagen even in the presence of MMP inhibitors. Importantly, the present study underlines the importance of taking into account the often-unappreciated phenomenon of spheroid expansion, which is affected by matrix features. Indeed, spheroid expansion was observed in pepsinized collagen gels but not in native collagen gels. Due to the aforementioned impact of spheroid expansion on the determination of global parameters, these observations recommend caution in the interpretation of data when using matrices composed of pepsinized collagen.

Despite being originally devised for the analysis of EC spheroids, the usefulness of the proposed method extends to a plethora of 3D-spheroid cultures that are currently used in different research fields to investigate the stemness of (cancer) cells [33] and/or the invasive properties of different cell types including cancer cells [34]–[36], leukocytes and trophoblastic cells [37]. Notably, the proposed methodology can be applied to characterise images of all-purpose spheroid assays.

Remarkably, our innovative method can be transposed to numerous applications of multicellular spheroid assays, including unravelling cell invasion mechanisms, investigating cell responses to pharmaceutical compounds and evaluating drug toxicity.
